# A prospective, randomized, double-blind, placebo-controlled, multicenter study of thiamin plus folic acid in the treatment of cognitive impairment in patients undergoing maintenance hemodialysis

**DOI:** 10.1080/0886022X.2026.2658981

**Published:** 2026-04-30

**Authors:** Kewei Xie, Yan Jiang, Tingfang Chen, Shang Liu, Yan Fang, Feihong Chen, Jianxiao Shen, Xiaojun Zeng, Ping Li, Ting Qiu, Jieying Wang, Ling Yu, Xiujuan Zang, Niansong Wang, Jiangzi Yuan, Huihua Pang, Weiming Zhang, Zhaohui Ni, Leyi Gu, Yongping Guo, Renhua Lu

**Affiliations:** ^a^ Department of Nephrology, Molecular Cell Lab for Kidney Disease, Shanghai Peritoneal Dialysis Research Center, Uremia Diagnosis and Treatment Center, Ren Ji Hospital, School of Medicine, Shanghai Jiao Tong University, Shanghai, China; ^b^ Department of Nephrology, Songjiang Hospital Affiliated to Shanghai Jiao Tong University School of Medicine, Shanghai, China; ^c^ Department of Nephrology, Sixth People’s Hospital, Shanghai Jiao Tong University, Shanghai, China; ^d^ Department of Nephrology, Baoshan Branch of Ren Ji Hospital, School of Medicine, Shanghai Jiao Tong University, Shanghai, China; ^e^ Clinical Center for Investigation, Ren Ji Hospital, School of Medicine, Shanghai Jiao Tong University, Shanghai, China; ^f^ Department of Neurology, Ren Ji Hospital, School of Medicine, Shanghai Jiao Tong University, Shanghai, China

**Keywords:** End-stage kidney disease, cognitive impairment, vitamin B, oxidative stress, cardiovascular outcomes

## Abstract

This prospective, randomized, double-blind, placebo-controlled trial investigated the efficacy and safety of thiamin and folic acid for cognitive impairment in maintenance hemodialysis (MHD) patients. A total of 215 MHD patients aged 18–75 with cognitive impairment were randomized to receive either oral thiamin (90 mg/day) plus folic acid (30 mg/day) or a placebo for 96 weeks. The primary endpoint was the change in the Alzheimer’s Disease Assessment Scale-Cognitive section (ADAS-Cog) score. After 96 weeks, the treatment group showed a significant improvement in ADAS-Cog scores (from 21.25 ± 9.2 to 15.07 ± 8.38, *p* < 0.001), whereas the placebo group showed a non‑significant improvement (from 24.53 ± 11.01 to 26.53 ± 14.43, *p* = 0.077). The treatment group also demonstrated significantly increased blood levels of thiamin (from 5.59 ± 0.95 to 18.21 ± 3.91 ng/mL) and folate (from 12.37 ± 4.62 to 63.33 ± 16.02 ng/mL), and a reduction in homocysteine levels (from 4709.06 ± 353.15 to 2962.68 ± 158.87 ng/mL, *p* < 0.001), with no significant changes in the placebo group. While mortality was similar between the two groups (12.1% vs. 12.0%, *p* = 0.978), the incidence of adverse events was significantly lower in the treatment group (31.8% vs. 62.0%, *p* = 0.0017), particularly cardiovascular and cerebrovascular events (13.1% vs. 25.9%, *p* = 0.001). The study concludes that combined thiamin and folic acid supplementation improves cognitive function in MHD patients with a favorable safety profile.

## Introduction

End-stage kidney disease (ESKD) is a public health issue of global concern, with a steadily increasing incidence. In addition to peritoneal dialysis and kidney transplantation, MHD is the main alternative treatment option for ESRD. The United States Renal Data System (USRDS) and the Chinese National Renal Data System (CNRDS) reported 475,142 and 751,098 patients undergoing MHD, respectively, by 2021 end, indicating increases of 1.2 and 3.2 times compared to 2011 [1,2]. CI is a common neurological complication of MHD [3]. Our previous study demonstrated that CI incidence in patients undergoing MHD is as high as 51.6%, and the 3-year survival rate is significantly lower in MHD cases with CI compared with counterparts with normal cognitive function [4]. This imposes a heavy burden on the country, society, and families, which requires widespread attention from the medical community.

Our previous *in vivo* and *in vitro* studies demonstrated that in 5/6 of nephrectomized rats, the uremic environment results in thiamin and folic acid deficiency, increased homocysteine levels, and enhanced oxidative stress in the hippocampal region of the brain, leading to neuronal damage and CI. In addition, the latter study found positive associations of serum thiamin and folic acid with cognitive function, which may be potential targets for the treatment in patients with CI undergoing MHD [5]. Recent findings also reported that thiamin, as a cofactor of transketolase, plays an important role in reducing reactive oxygen species (ROS) production in the nervous system and alleviating oxidative stress [6]. Folic acid exerts a direct antioxidant effect and is an essential substance for the metabolism of homocysteine to methionine, which reduces homocysteine levels [7]. Based on the above findings, a prospective, randomized, controlled, open-label and single-center clinical study was performed, including 50 patients with CI undergoing MHD. Of these patients,25 each were included in the treatment (thiamin tablets 90 mg/day plus folic acid tablets 30 mg/day) and control (without intervention) groups, with a follow-up of 96 weeks.

The results revealed a significant decrease of 25.2% in blood homocysteine levels and a significant improvement of the MoCA score in the treatment group. However, the latter study had several limitations, including a small sample size, the absence of blinding and placebo control methods, and the use of only the MoCA score to assess cognitive function [8].

To further validate the above preliminary trial, a prospective, randomized, double-blind, placebo-controlled multicenter clinical study [9] was designed to examine the efficacy and safety of two B vitamin-intensive combination therapies for improving cognitive function in patients with CI undergoing MHD.

## 2. Patients and methods

### 2.1. Patients

**Figure 1.**
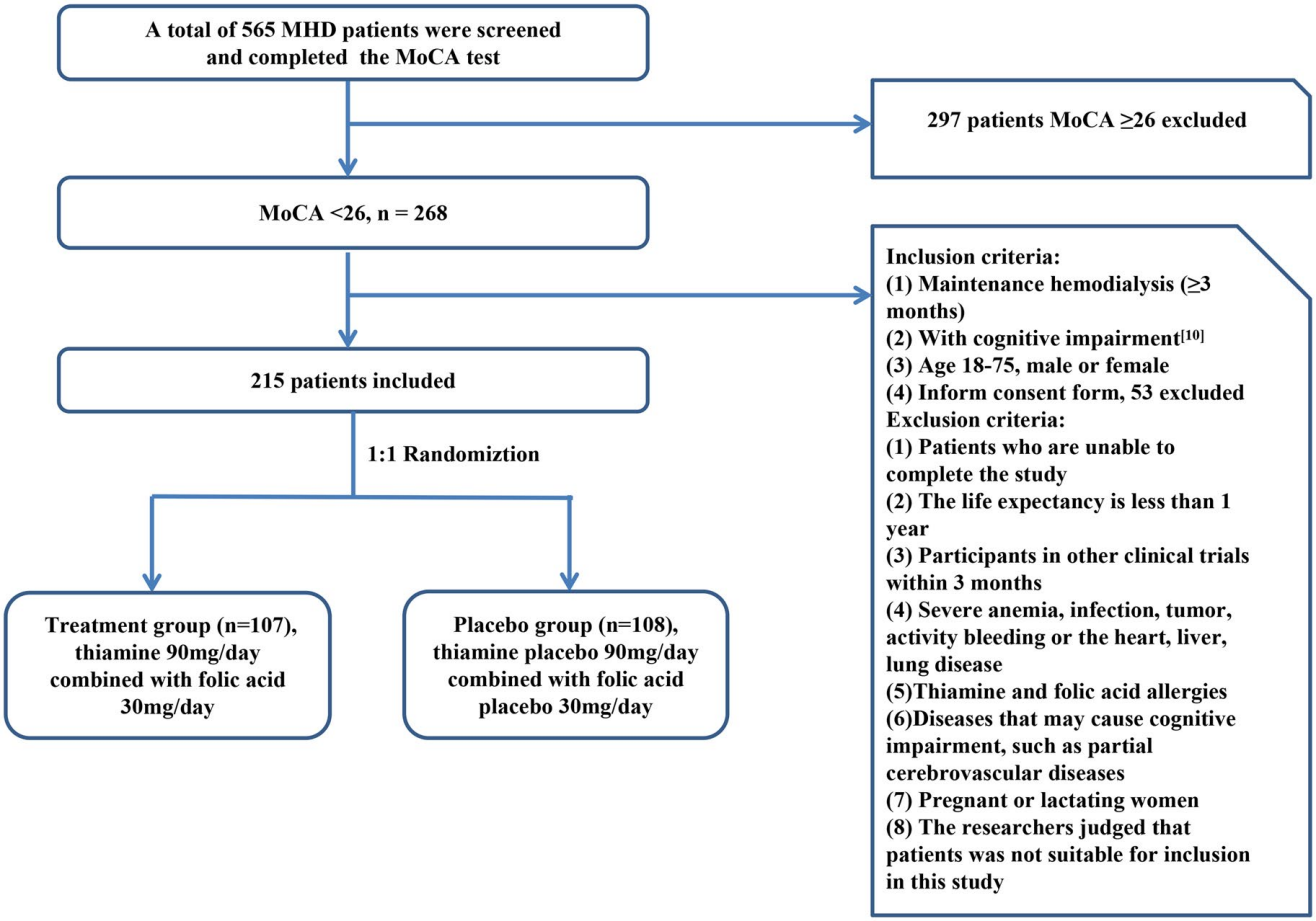
Patient inclusion and exclusion criteria. According to the inclusion and exclusion criteria, a total of 215 maintenance hemodialysis patients entered the randomized trial, Treatment Group (*n* = 107), Placebo Group (*n* = 108).

Patients undergoing maintenance hemodialysis at Renji Hospital Affiliated to Shanghai Jiao Tong University School of Medicine, Songjiang Hospital Affiliated to Shanghai Jiao Tong University School of Medicine, Shanghai Sixth People’s Hospital, and Baoshan Branch of Renji Hospital Affiliated to Shanghai Jiao Tong University School of Medicine were screened according to set inclusion and exclusion criteria (Figure 1).

### 2.2. Methods

#### 2.2.1. Study design

This was a prospective, randomized, double-blind, placebo-controlled, multicenter clinical study. The current study was registered with the Chinese Clinical Trial Registry (https://www.chictr.org.cn/index.html, Date of Registration: 2020-01-22, Registration number: ChiCTR2000029297). This clinical trial was conducted from January 18, 2021, to April 30, 2023.

#### 2.2.2. Randomization and double-blinding methods

A total of 215 patients were consecutively enrolled between January and April 2021. Random sequences were generated with SAS (version 9.3) by an independent data manager, who printed the random numbers and stored them in sealed opaque envelopes. A centralized 1:1 randomization was applied. Stratified randomization was performed based on three key factors: study center, age (<60 vs. ≥60 years), and dialysis vintage (<36 vs. ≥36 months), to ensure balanced distribution of potential confounding variables between groups. Each time a patient signed the informed consent form and met the inclusion criteria, the study doctor opened a random envelope to obtain a random number based on which the corresponding number of study drugs was assigned to the subject.

#### 2.2.3. Patient grouping

The treatment group was administered thiamin tablets (90 mg/day,30 mg tid po, specification 10 mg/tablet,100 tablets/bottle) and folic acid tablets (30 mg/day,10 mg tid po, specification 5 mg/tablet, 100 tablets/bottle). The placebo group was administered thiamin (90 mg/day, 30 mg tid po, Shanghai Chenpon Pharmaceutical Co., Ltd., specification 10 mg/tablet, 100 tablets/bottle) and folic acid (30 mg/day,10 mg tid po, Changzhou Pharmaceutical Factory, specification 5 mg/tablet,100 tablets/bottle) placebos.

#### 2.2.4. Drug management

Each branch center managed the trial drugs using the same procedure, distributing 3 bottles of thiamin or placebo tablets and 2 bottles of folic acid or placebo tablets every 4 weeks. Before each distribution, the drug administrator collected the remaining drugs from the previous distribution and filled out the Drug Distribution and Recovery Form. Meanwhile, all subjects filled out the Subject Diary.

### 2.3. Efficacy evaluation indicators

The primary efficacy endpoint was Alzheimer’s disease assessment scale-cognitive section (ADAS-Cog) score, which was assessed in the treatment and placebo groups at baseline and Week 96. Secondary efficacy endpoints included changes in blood thiamin, folic acid, and homocysteine levels in the treatment and placebo groups at baseline and Week 96, as well as patient prognosis in both groups at 96 weeks. Safety endpoints were changes in laboratory safety indicators and the incidence of AEs during the study period.

### 2.4. Main outcome measures

#### 2.4.1. Cognitive function assessment

At baseline, the MoCA score [10,11] was employed to diagnose CI (<26 points [<25 points in case of <12 educational years]). The version of the MoCA scoring system was the Beijing version (2006-8-26). At baseline, 48 weeks of follow-up, and 96 weeks of follow-up, the ADAS-Cog score [12,13] was applied to assess treatment efficacy. Both assessments were performed by trained neurologists before or within 1 h of hemodialysis. The randomization data were kept confidential by neurologists performing cognitive function assessments.

#### 2.4.2. Blood thiamin, folic acid, and homocysteine detection

Blood thiamin, folic acid, and homocysteine levels were measured at baseline and 48 and 96 weeks of follow-up. Ultra-high performance liquid chromatography tandem quadrupole mass spectrometry (API 4000 triple quadrupole liquid chromatography-mass spectrometer, AB Sciex Analytical Instrument Trading Co., Ltd.) was performed, with Analyst 1.61 for analysis.

### 2.5. Statistical analysis

Efficacy and safety analyses will be performed according to the Intention-To-Treat (ITT) principle on all randomized subjects who received at least one dose of the study medication, using the Last Observation Carried Forward (LOCF) method to impute missing data for subjects who did not complete the full treatment course. Normally distributed continuous data will be presented as Mean ± Standard Deviation (SD), non-normally distributed continuous data as Median and Interquartile Range (IQR), and categorical data as Proportions or Percentages (%). Quantitative data will be analyzed using the t-test or Wilcoxon rank-sum test, while qualitative data will be analyzed using the Chi-square test, Fisher’s exact test, or Cochran-Mantel-Haenszel (CMH) chi-square test. Baseline comparisons between groups will utilize the Mann-Whitney U test (non-parametric test). Due to differences in sex composition between the two groups at baseline, 2 × 3 × three factor repeated measures ANOVA was used to analyze cognitive scores. Among them, group (placebo group, treatment group) and biologic sex (male, female) were used as intergroup factors, and time (baseline, 48 weeks, 96 weeks) was used as intra group factors. This model is used to examine the interaction between groups and time (i.e. evaluate whether treatment response patterns vary by group), while controlling for the main effect of sex and all possible interactions. If the high-order interaction is not significant, it will be removed from the final model. Survival analysis will employ Kaplan-Meier survival curves. All statistical analyses will be conducted using the SPSS version 27.0 software package (SPSS Inc., Chicago, IL, USA), with a *p*-value < 0.05 defined as statistically significant.

## 3. Results

### 3.1. Basic information of patients undergoing MHD

**Figure 2.**
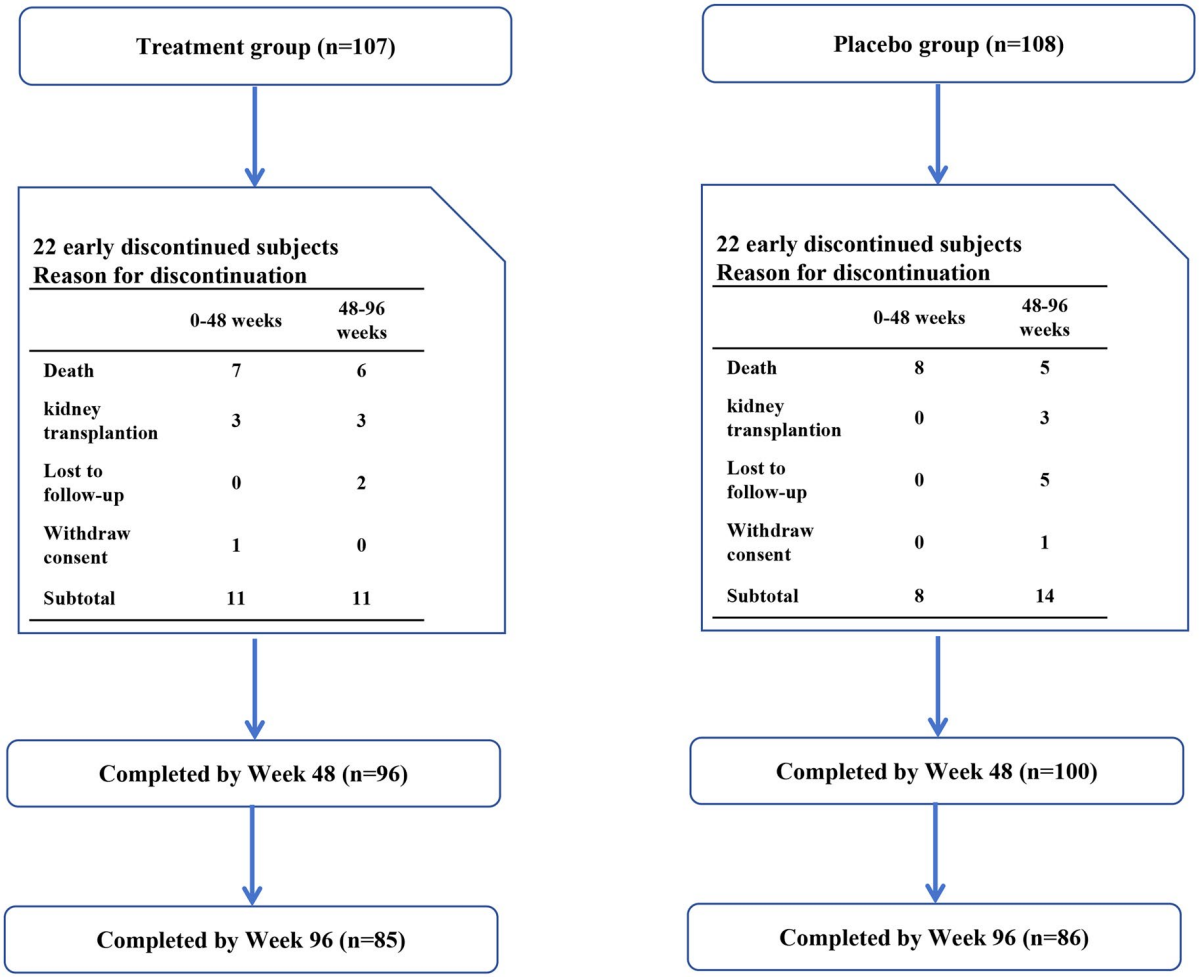
Patient disposition from randomiztion to end of study. This study was followed up for 96 weeks, with 96 (Treatment Group) and 100 patients (Placebo Group) completing 48 weeks of follow-up, while 85 (Treatment Group) and 86 (Placebo Group)patients completed 96 weeks of follow-up, respectively. Both groups had 22 patients who did not complete 96 weeks of follow-up.

A total of 215 patients undergoing MHD met the inclusion and exclusion criteria. According to the study protocol, the patients were randomly divided into the treatment (*n* = 107) and placebo (*n* = 108) groups at a ratio of 1:1, with a follow-up period of 96 weeks (Figure 2).

There were no statistically significant differences in baseline demographic data, past history, and cognitive function scores (MoCA and ADAS-Cog scores) between the treatment and placebo groups, except for a significantly lower proportion of male patients in the treatment group versus the placebo group. The proportion of male patients was significantly lower in the treatment group (54.2%) than in the placebo group (68.5%, *p* = 0.036). There were also no significant differences in baseline hemodialysis-related data and laboratory indicators between the treatment and placebo groups (Table 1).

**Table 1.** Characteristics of the patients at baseline.

	Total (*n* = 215)	Treatment group (*n* = 107)	Placebo group (*n* = 108)	*P* Value
Age, years	59.12 ± 11.82	58.39 ± 13.12	59.84 ± 10.37	0.370
Male, *n* (%)	132 (61.4)	58 (54.2)	74 (68.5)	0.036
Height (cm)	166.63 ± 7.24	166.53 ± 7.0	166.73 ± 7.50	0.842
Body Mass Index (BMI)	21.97 ± 3.15	22.17 ± 3.33	21.77 ± 2.97	0.390
Education status (≥12 years, *n* [%])	89 (41.4)	42 (39.3)	47 (43.5)	0.793
Primary cause of MHD, *n* (%)				
Primary glomerulonephritis	73 (34)	32 (29.9)	41 (38)	0.250
Diabetic nephropathy	33 (15.3)	12 (11.2)	21 (19.4)	0.129
Hypertensive nephrosclerosis	8 (3.7)	3 (2.8)	5 (4.6)	0.721
ADPKD	17 (7.9)	11 (10.3)	6 (5.6)	0.218
Unknown cause	84 (39)	45 (42)	39 (36.2)	0.851
Complication, *n* (%)				
Hypertension	141 (65.6)	68 (63.6)	73 (67.6)	0.568
Diabetes	61 (28.40	27 (25.2)	34 (31.5)	0.365
Cardiovascular disease	25 (11.6)	13 (12.1)	12 (11.1)	0.835
Cerebrovascular disease	11 (5.1)	4 (3.7)	7 (6.5)	0.538
Urine, mL/day	0 (0, 100)	0 (0, 100)	0 (0, 100)	0.803
Dialysis vintage, months	40.5 (17, 97.75)	40 (19, 99)	41 (16.5, 94.5)	0.711
MoCA Score	19.65 ± 5.06	19.40 ± 5.40	19.94 ± 4.65	0.454
ADAS-Cog Score	22.93 ± 10.98	22.19 ± 10.31	23.65 ± 11.61	0.327
Autogenous arteriovenous fistula (patients, %)	211 (98.1)	105 (98.1)	106 (98.1)	1.000
Intradialytic hypotension (patients, %)	17 (7.9)	9 (8.4)	8 (7.4)	0.594
Pre-dialysis weight (Kg)	62.88 ± 10.22	63.62 ± 11.23	62.16 ± 10.62	0.336
Post-dialysis weight (Kg)	60.86 ± 10.83	61.68 ± 11.11	60.06 ± 10.54	0.283
Ultrafiltration (mL)	2407.63 ± 817.18	2438.5 ± 754.5	2377.3 ± 876.9	0.587
Pre-dialysis systolic pressure (mmHg)	143.94 ± 24.86	144.26 ± 23.17	143.63 ± 26.56	0.854
Post-dialysis systolic pressure (mmHg)	136.27 ± 24.44	136.87 ± 25.1	135.68 ± 23.86	0.725
Pre-dialysis diastolic pressure (mmHg)	76.79 ± 17.12	77.24 ± 14.9	76.33 ± 19.15	0.703
Post-dialysis diastolic pressure (mmHg)	77.4 ± 14.81	78.91 ± 15.71	75.9 ± 13.76	0.139
Hb (g/L)	112.01 ± 12.34	113.18 ± 12.05	110.94 ± 12.56	0.201
Alb (g/L)	40.06 ± 2.97	40.27 ± 3.23	39.86 ± 2.70	0.318
Glu (mmol/L)	7.29 ± 3.72	6.98 ± 3.70	7.6 ± 3.73	0.230
Glycated hemoglobin (%)	5.77 ± 1.07	5.61 ± 0.94	5.96 ± 1.19	0.058
TC (mmol/L)	4.14 ± 1.06	4.23 ± 1.12	4.05 ± 0.99	0.217
TG (mmol/L)	2.01 ± 1.39	2.1 ± 1.64	1.92 ± 1.09	0.350
LDL (mmol/L)	2.48 ± 0.85	2.53 ± 0.91	2.42 ± 0.78	0.373
HCO_3_^−^ (mmol/L)	22.18 ± 3.2	21.75 ± 3.43	22.62 ± 2.87	0.113
K^+^ (mmol/L)	4.56 ± 1.09	4.57 ± 0.98	4.58 ± 1.11	0.954
Ca (mmol/L)	2.22 ± 0.42	2.27 ± 0.45	2.17 ± 0.38	0.082
P (mmol/L)	1.99 ± 0.67	1.97 ± 0.66	2.0 ± 0.68	0.755
TSAT (%)	27.89 ± 12.41	28.32 ± 11.63	27.45 ± 13.22	0.636
Ferritin (ng/mL)	117(42.75, 357)	154(42, 373)	104(43, 340.5)	0.452
iPTH (pg/mL)	337.82 ± 286.26	315.52 ± 277.92	359.06 ± 293.74	0.277
25-(OH)-D3 (mIU/L)	12.52 ± 5.92	12.71 ± 5.36	12.32 ± 6.52	0.754
CRP (mg/dL)	2(0, 5)	2(0, 4)	4.26(1.77, 7.77)	0.425
β_2_-MG (mg/L)	26.99 ± 13.85	27.67 ± 14.49	26.29 ± 13.21	0.481
BNP (pg/mL)	598(183, 2676.25)	448.5(183, 2540)	686(185.75, 2890.75)	0.377
spKt/V	1.43 ± 0.32	1.47 ± 0.31	1.39 ± 0.33	0.110

### 3.2. ADAS-cog scores at baseline and Week 96

**Figure 3.**
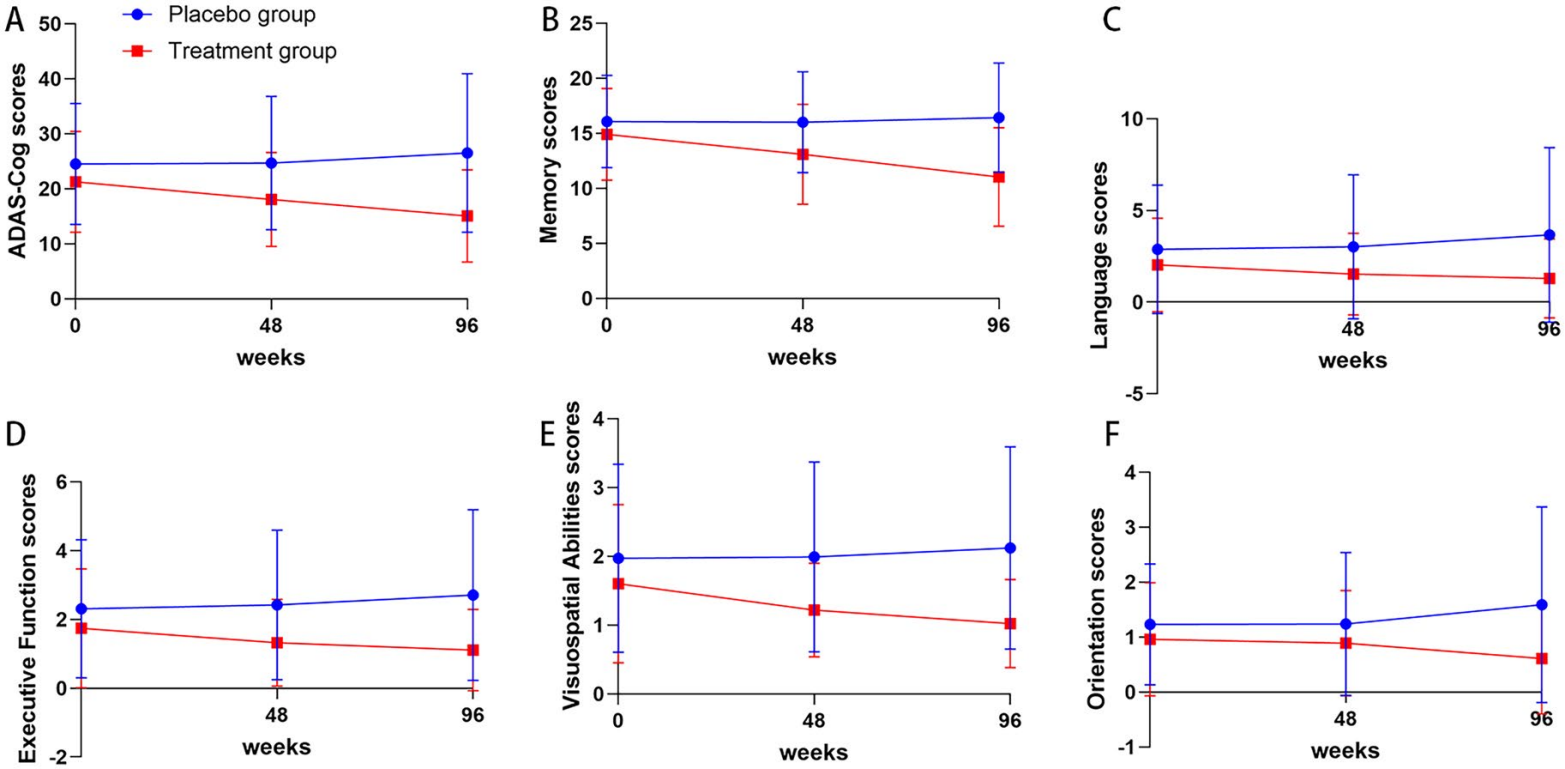
Comparison of ADAS-Cog Scores Between Treatment and Placebo Groups: Based on ADAS-Cog scores, the treatment group demonstrated improved cognitive impairment compared to the placebo group, with a statistically significant difference in ADAS-Cog scores between the two groups at 96 weeks. Panels A–F represent the ADAS-Cog total score and domain scores for memory function, language ability, executive function, visuospatial ability, and orientation, respectively.

After 96 weeks of follow-up, ADAS-Cog scores were significantly lower in the treatment group than in the control group. Meanwhile, ADAS-Cog scores in the treatment group (*n* = 85) were significantly lower than baseline values. At Week 96, ADAS-Cog scores in the placebo group (*n* = 86) showed an upward trend compared with baseline values, but without statistical significance (Figure 3(A)). The interaction between group and time was significant (*F* = 41.25, *p* < 0.001), indicating a significant difference in the pattern of cognitive score changes over time between the two groups of patients, supporting the therapeutic effect of the drug. The third-order interaction between group, sex, and time was not significant (*F* = 0.007, *p* = 0.993), and the interaction between time and sex was also not significant (*F* = 0.097, *p* = 0.908). This indicates that sex differences did not significantly alter the pattern of cognitive score changes over time, nor did they modulate the efficacy pattern of drugs. Therefore, subsequent analysis will focus on significant group time interactions (Table 2).

**Table 2.** Comparison of ADAS-cog scores between two groups of maintenance hemodialysis patients at different follow-up time points.

Indicator (ADAS-Cog Scores)	Control group (*n* = 86)	Treatment group (*n* = 85)	*Z*-value	*p*-value
0 week	24.53 ± 11.01	21.25 ± 9.20	1.071	0.301
48 weeks	24.68 ± 12.12	18.07 ± 8.57	18.467	<0.001
96 weeks	26.53 ± 14.43	15.07 ± 8.38	48.329	<0.001
F-value	2.638	98.87		
*p*-value	0.077	<0.001		
Between-group effect				
Group	*F* = 20.183, *p* < 0.001			
Sex	*F* = 0.040, *p* = 0.842			
Group × Sex	*F* = 0.408, *p* = 0.524			
Within-group effect				
Time	*F* = 11.463, *p* < 0.001			
Time × Group	*F* = 41.250, *p* < 0.001			
Time × Sex	*F* = 0.097, *p* = 0.908			
Time × Group × Sex	*F* = 0.007, *p* = 0.993			
Indicator (memory scores)	Control group (*n* = 86)	Treatment group (*n* = 85)	Z-value	*p*-value
0 week	16.07 ± 4.18	14.91 ± 4.18	0.578	0.447
48 weeks	16.01 ± 4.59	13.09 ± 4.54	16.421	<0.001
96 weeks	16.43 ± 4.97	11.04 ± 4.49	44.254	<0.001
F-value	1.148	80.87		
*p*-value	0.322	<0.001		
Between-group effect	*F* = 24.073, *p* < 0.001			
Within-group effect	*F* = 26.229, *p* < 0.001			
Group × Time interaction	*F* = 40.25, *p* < 0.001			
Indicator (Language scores)	Control group (*n* = 86)	Treatment group (*n* = 85)	Z-value	*p*-value
0 week	2.88 ± 3.5	2.03 ± 2.55	2.329	0.127
48 weeks	3.02 ± 3.93	1.53 ± 2.22	12.339	<0.001
96 weeks	3.67 ± 4.77	1.29 ± 2.15	35.722	<0.001
F-value	2.979	12.558		
*p*-value	0.056	<0.001		
Between-group effect	*F* = 10.65, *p* = 0.001			
Within-group effect	*F* = 3.413, *p* = 0.035			
Group × Time interaction	*F* = 11.037, *p* < 0.001			
Indicator (Executive Function Scores)	Control group (*n* = 86)	Treatment group (*n* = 85)	Z-value	*p*-value
0 week	2.31 ± 2.01	1.74 ± 1.73	3.008	0.083
48 weeks	2.42 ± 2.18	1.32 ± 1.26	13.799	<0.001
96 weeks	2.71 ± 2.48	1.11 ± 1.18	38.624	<0.001
F-value	2.932	12.331		
*p*-value	0.059	<0.001		
Between-group effect	*F* = 1.448, *p* < 0.001			
Within-group effect	*F* = 2.45, *p* = 0.089			
Group × Time interaction	*F* = 12.081, *p* < 0.001			
Indicator (Visuospatial Abilities Scores)	Control group (*n* = 86)	Treatment group (*n* = 85)	Z-value	*p*-value
0 week	1.97 ± 1.37	1.6 ± 1.15	1.871	0.171
48 weeks	1.99 ± 1.38	1.22 ± 0.68	17.221	<0.001
96 weeks	2.12 ± 1.47	1.02 ± 0.64	40.291	<0.001
F-value	1.798	17.208		
*p*-value	0.172	<0.001		
Between-group effect	*F* = 19.67, *p* < 0.001			
Within-group effect	*F* = 6.697, *p* = 0.002			
Group × Time interaction	*F* = 15.133, *p* < 0.001			
Indicator (Orientation Scores)	Control group (*n* = 86)	Treatment group (*n* = 85)	Z-value	*p*-value
0 week	1.23 ± 1.1	0.96 ± 1.03	2.225	0.136
48 weeks	1.24 ± 1.30	0.89 ± 0.96	3.044	0.081
96 weeks	1.59 ± 1.78	0.61 ± 1.01	30.609	<0.001
F-value	2.699	20.539		
*p*-value	0.073	<0.001		
Between-group effect	*F* = 10.469, *p* = 0.001			
Within-group effect	*F* = 0.12, *p* = 0.851			
Group × Time interaction	*F* = 8.507, *p* < 0.001			

Based on significant between-group differences in overall cognitive function (ADAS-Cog total score) (between-group effect *F* = 20.306, *p* < 0.001; interaction effect *F* = 29.239, *p* < 0.001), the scores were categorized into five cognitive domains for analysis: memory function, language ability, executive function, visuospatial ability, and orientation. Results showed that the treatment group demonstrated continuous and significant improvements in all cognitive domains (all *p* < 0.001). At week 96, significant decreases from baseline were observed in memory function (26.0% reduction), language ability (36.5% reduction), executive function (36.2% reduction), visuospatial ability (36.3% reduction), and orientation (36.5% reduction), with particularly prominent improvements in memory function (within-group *F* = 80.87) and orientation (within-group *F* = 20.54). In contrast, the control group exhibited only marginally significant deterioration trends in language ability (*p* = 0.056) and executive function (*p* = 0.059), while the remaining domains remained stable (all *p* > 0.05). Significant interaction effects were observed across all domains (all *p* < 0.001, strongest in the memory domain *F* = 40.25), confirming that the therapeutic intervention comprehensively slowed cognitive decline in maintenance hemodialysis patients, with particularly significant effects on memory improvement, as detailed in Table 2 and Figure 3(B–F).

### 3.3. Blood thiamin, folic acid, and homocysteine levels at baseline and Week 96

There were no significant differences in blood thiamin, folic acid, and homocysteine levels between the two groups at baseline. After 96 weeks of follow-up, blood homocysteine levels were significantly lower in the treatment group compared with the placebo group. Meanwhile, blood thiamin and folic acid levels were significantly higher in the treatment group compared with the placebo group (Table 3).

**Table 3.** Secondary outcomes.

	Treatment group	Placebo group	*P* Value
Secondary outcomes			
Thiamin (ng/mL)			
Baseline (n)	5.59 ± 0.95(107)	5.03 ± 0.72(108)	0.637
48 Weeks (n)	18.84 ± 2.68(96)	5.08 ± 0.95(100)	<0.001
96 Weeks (n)	18.21 ± 3.91(85)	3.31 ± 0.28(86)	<0.001
Comparison between 96 weeks and baseline, *p* value	0.003	0.037	
Folic acid (ng/mL)			
Baseline (n)	12.37 ± 4.62(107)	8.44 ± 3.07(108)	0.478
48 weeks (n)	62.15 ± 10.2(96)	7.74 ± 0.52(100)	<0.001
96 weeks (n)	63.33 ± 16.02(85)	7.63 ± 0.08(86)	<0.001
Comparison between 96 weeks and baseline, *p* value	<0.001	0.008	
Homocysteine (ng/mL)			
Baseline (n)	4709.06 ± 353.15(107)	5440.45 ± 336.33(108)	0.135
48 weeks (n)	3777.02 ± 1230.8(96)	5484.09 ± 513.91(100)	0.003
96 weeks (n)	2962.68 ± 158.87(85)	5158.17 ± 361.87(86)	<0.001
Comparison between 96 weeks and baseline, *p* value	<0.001	0.648	
Death (n, %)	13(12.1%)	13(12.0%)	0.978
Cardiovascular and cerebrovascular events, n (%)	9(8.4%)	7(6.5%)	
Infection, n (%)	3(2.8%)	5(4.6%)	
Malignant tumor, n (%)	0(0)	1(0.9%)	
Gastrointestinal bleeding, n (%)	1(0.9%)	0(0%)	

In addition, at 48 and 96 weeks of follow-up, blood thiamin and folic acid levels were significantly higher in the treatment group versus baseline values, while blood homocysteine levels were significantly lower than baseline values, with decrease rates of 19.79% and 37.09%, respectively. Blood thiamin and folic acid levels were significantly lower in the placebo group at 96 weeks of follow-up versus baseline values, while no significant differences were found in blood homocysteine levels between 48 and 96 weeks (Table 3).

### 3.4. Patient prognosis at 96 weeks of follow-up

**Figure 4.**
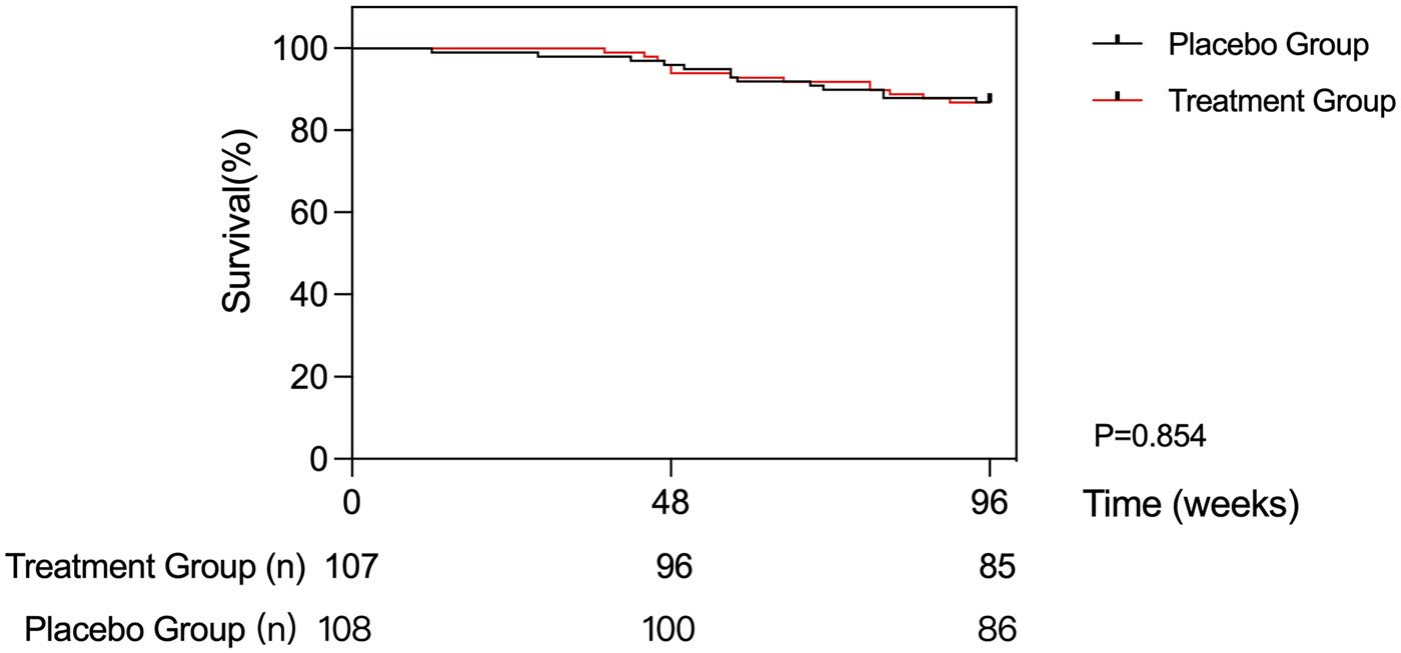
Survival curve analysis. Kaplan–Meier survival analysis also revealed no significant difference in survival rate between the treatment and placebo groups (*p* = 0.854).

During the follow-up period, a total of 26 patients died, including 13 each in the treatment (12.1%) and placebo (12%) groups, indicating no significant difference between the two groups (*p* = 0.978). Kaplan-Meier survival analysis also revealed no significant difference in survival rate between the treatment and placebo groups (*p* = 0.854), as shown in Figure 4. In addition, 9 patients in each group did not complete the 96 weeks of follow-up. Of these, 6 patients in the treatment group had kidney transplantation surgery (5.6%), 2 were transferred to another center (1.9%), and 1 (0.9%) requested to withdraw from the study. In the placebo group, 3 patients had kidney transplantation (2.8%), 5 were transferred to another center (4.6%), and 1 requested to withdraw from the study (0.9%). There was no significant difference between the two groups (*p* = 0.683), as shown in Figure 2.

The major causes of death were cardiovascular and cerebrovascular diseases (16 cases, 61.5%), infections (8 cases, 23.1%), malignant tumors (1 case, 3.8%), and gastrointestinal bleeding (1 case, 3.8%) (Table 4). There was no significant difference (*p* = 0.688) between the treatment and placebo groups in the 16 patients who died from cardiovascular and cerebrovascular diseases.

**Table 4.** Adverse events.

	Treatment group (*n* = 107)	Placebo group (*n* = 108)	*p*-value
Cardiovascular and cerebrovascular events, n (%)	14 (13.1%)	28 (25.9%)	0.001
Vascular access events, n (%)	4 (3.7%)	10 (9.3%)	0.089
Infection, n (%)	5 (4.7%)	9 (8.3%)	0.125
Covid-19, n (%)	3 (2.8%)	4 (3.7%)	1.000
Gastrointestinal events, n (%)	5 (4.7%)	7 (6.5%)	0.357
Malignant tumor, n (%)	0 (0)	2 (1.9%)	0.845
Other events, n (%)	6 (5.6%)	11 (10.2%)	0.108
Total, n (%)	34 (31.8%)	67 (62%)	0.0017

### 3.5. Changes in laboratory safety indicators and incidence of AEs during the study period

Compared with the placebo group, there were no significant differences in hemodialysis-related data and laboratory indicators between the treatment and placebo groups at 48 and 96 weeks of follow-up, except for significantly lower blood phosphorus levels in the treatment group compared with the placebo group at Week 96 (see Supplementary Table).

During the study period, a total of 101 AEs occurred, including 34 (33.7%) and 67 (66.3%) in the treatment and placebo groups, respectively. The incidence of AEs was significantly higher in the placebo group compared with the treatment group (*p* = 0.0017). During the follow-up period, a total of 42 cardiovascular and cerebrovascular AEs occurred, including 14 and 28 in the treatment and placebo groups, respectively, indicating a significant difference between the two groups (*p* = 0.001). Seven patients had novel coronavirus pneumonia during the study period, including 3 and 4 in the treatment and placebo groups, respectively, indicating no statistically significant difference between the two groups (*p* = 1.000) (Table 3).

## 4. Discussion

This study employed a prospective, randomized, double-blind, placebo-controlled design. The results showed that after 96 weeks of follow-up, patients in the treatment group exhibited a significant reduction in ADAS-Cog scores, indicating that supplementation with thiamin and folic acid can improve cognitive function in MHD patients with CI. Simultaneously, serum homocysteine levels were significantly lower in the treatment group than in the placebo group, while serum thiamin and folic acid levels were significantly higher. Furthermore, the incidence of adverse events, particularly cardiovascular and cerebrovascular events, was significantly higher in the placebo group than in the treatment group. However, there was no significant difference in survival rates between the two groups.

The primary finding of this study is that after 96 weeks of treatment with thiamin (90 mg/day) combined with folic acid (30 mg/day), patients in the treatment group had significantly lower ADAS-Cog scores than those in the placebo group, suggesting improved cognitive function. Thiamin and folic acid deficiency have been confirmed to induce increased oxidative stress in the cerebral cortex and hippocampus, leading to neuronal damage and cognitive impairment, a conclusion supported by *in vivo* and *in vitro* studies [5,14–16]. Recent studies indicate that B vitamin supplementation (including thiamin and folic acid) can effectively slow cognitive decline and neurodegeneration in the elderly Chinese population [17] and improve cognitive function in Alzheimer’s disease patients [18]. The underlying mechanisms include: Thiamin acts as an oxygen free radical scavenger, reducing the production of reactive oxygen species (ROS) in the nervous system and alleviating hypermethylation of redox-related genes NUDT15 and TXNRD1, thereby mitigating oxidative stress [14] and oxidative damage [19], and improving cognitive function; Folic acid has direct antioxidant effects, inhibiting inflammatory cytokine expression and alleviating oxidative stress [7,18] by reducing S-adenosylhomocysteine (SAH) levels and increasing S-adenosylmethionine (SAM) levels, consequently enhancing cognitive ability. Animal experiments further found that folic acid supplementation improves cognitive function by increasing thymidylate synthase (TS) expression in SAMP8 (Senescence-Accelerated Mouse Prone 8), thereby reducing DNA damage and neuronal apoptosis [20]. Cognitive domain-specific analysis further revealed that this combined intervention particularly significantly improved memory and executive function in MHD patients: The hippocampus (the memory center) is highly sensitive to uremic toxins and hyperhomocysteinemia [21], and the folic acid pathway specifically protects memory function by promoting homocysteine remethylation, reducing oxidative stress and abnormal tau protein phosphorylation [22]; The prefrontal cortex (executive function) is impaired due to thiamin-dependent glycolytic dysfunction, which thiamin corrects by restoring neuronal mitochondrial ATP synthesis and enhancing dopaminergic signaling [23,24]. Together, they form a synergistic ‘metabolic-synaptic protection’ effect, while brain regions related to visuospatial ability/orientation are relatively less sensitive to this vitamin intervention.

In the chronic kidney disease (CKD) population, our previous small-sample pilot study also found that 96 weeks of combined thiamin and folic acid treatment improved Montreal Cognitive Assessment (MoCA) scores in MHD patients with CI [8]. However, a limitation of that study was that it only used MoCA scores to assess cognitive function before and after treatment. Previous literature points out that in cognitive function research, MoCA scores are primarily used for diagnosing cognitive impairment, while ADAS-Cog scores are the standard metric for assessing cognitive improvement following pharmacotherapy [25]. This study employed ADAS-Cog scores as the efficacy evaluation criterion, addressing the limitations of the previous study and further validating that thiamin combined with folic acid improves cognitive function in MHD patients with CI. It is noteworthy that cognitive impairment is now considered a hard endpoint indicator for the prognosis of MHD patients [26], meaning that improving cognitive function may improve prognosis; the results of this study provide an important basis for future in-depth research.

This study found that patients in both groups had serum thiamin and folic acid deficiency at baseline, with levels lower than those in the normal population. This result is consistent with our previous animal study [5] and small-sample pilot experiment [8]. The reasons may be related to the fact that thiamin and folic acid, as water-soluble vitamins, have their absorption affected by uremic toxins [27]; additionally, they are cleared during MHD, especially when receiving high-flux dialysis. Some studies have reported that high-dose thiamin (100 mg tid × 7 days, then 100 mg qd) and folic acid (40 mg qd) supplementation can improve cognitive function and reduce the risk of cardiovascular and cerebrovascular events [28]. The results of this study show that after 96 weeks of supplementation with thiamin (90 mg/day) combined with folic acid (30 mg/day), serum thiamin and folic acid levels in the treatment group were significantly higher than baseline. However, serum thiamin levels remained below the normal range (22.4–57.6 ng/mL) [29], which may be related to the relatively low therapeutic dose and the downregulation of thiamin transporter 1 (THTR1) and thiamin transporter 2 (THTR2) expression caused by the uremic environment [27]. This suggests that the current dose, while effective for cognitive improvement, may be suboptimal for fully correcting thiamin deficiency in MHD patients. Future prospective dose-finding studies are needed to establish the optimal therapeutic dose that normalizes thiamin levels while maximizing clinical benefits. whereas serum folic acid levels were higher than the range for the general population (2.7–17 ng/mL), a phenomenon consistent with the pilot results [8]. Notably, serum homocysteine levels decreased significantly in the treatment group, with the increase in serum folic acid levels presumed to play a major role. However, the significant increase in serum thiamin levels and its contribution to alleviating oxidative stress, reducing homocysteine levels, and improving cognitive function should not be overlooked. Although the dose used in this study improved cognitive function, future prospective studies are needed to explore the optimal therapeutic dose for MHD patients with CI to achieve better survival outcomes.

Recent studies indicate that elevated serum homocysteine levels are closely associated with cognitive impairment [30,31]. In MHD patients, the mechanism for its elevation includes B vitamin deficiency (especially thiamin and folic acid) [32]. In this study, baseline serum homocysteine levels in both groups were significantly higher than the normal range, indicating the presence of oxidative stress [33]. After 96 weeks of intervention, serum homocysteine levels in the treatment group decreased significantly (a reduction of 37.09%). Literature reports that oxidative stress is one of the common pathogenic mechanisms for cognitive impairment and cardiovascular/cerebrovascular diseases in MHD patients [3], and a reduction in homocysteine levels >20%, as an indicator of oxidative stress, can significantly reduce the risk of adverse cardiovascular events [34]. This is consistent with the observation in this study that the incidence of adverse cardiovascular/cerebrovascular events was significantly lower in the treatment group than in the placebo group. Although cardiovascular/cerebrovascular disease is the leading cause of death in MHD patients, this study found no statistically significant difference in overall survival rates between the two groups, which may be related to the relatively small sample size, limited number of mortality events, and short follow-up period, which constrained the statistical power to detect a survival difference. Future randomized controlled trials (RCTs) with larger sample sizes, longer follow-up, and patient prognosis as the primary endpoint are needed for verification.

This study has the following limitations: 1. It primarily relied on ADAS-Cog scores to assess cognitive function, which is subjective; future studies need to incorporate more objective assessment indicators (such as functional magnetic resonance imaging [fMRI] [35]); 2. The combination therapy design makes it difficult to distinguish the independent effects of thiamin and folic acid. Future studies with a factorial design are warranted to elucidate their individual contributions; 3. The optimal therapeutic dose for the two drugs was not explored.

In conclusion, supplementation with thiamin (90 mg/day) combined with folic acid (30 mg/day) can safely and effectively improve cognitive function and reduce the risk of adverse cardiovascular/cerebrovascular events in MHD patients with CI.

## Supplementary Material

Supplement Table 1.docx

## Data Availability

All data generated and described in this article are available from the corresponding author on reasonable request.
