# Supplementary material for: A prospective, randomized, double-blind, placebo-controlled, multicenter study of thiamin plus folic acid in the treatment of cognitive impairment in patients undergoing maintenance hemodialysis
Source: Ren Fail. 2026 Apr 30;48(1):2658981. doi: 10.1080/0886022X.2026.2658981 (PMC13134739; doi:10.1080/0886022X.2026.2658981)
Supplement: Supplement Table 1.docx [file IRNF_A_2658981_SM4572.docx]

**Supplement Table. Comparison of hemodialysis data and laboratory indicators in the the treatment group and the placebo group at 48 and 96 weeks of follow-up.**

|  | Treatment group | | Placebo group | |
| --- | --- | --- | --- | --- |
|  | 48 Weeks  (n=96) | 96 Weeks  (n=85) | 48 Weeks  (n=100) | 96 Weeks  (n=86) |
| Autogenous arteriovenous fistula (patients, %) | 93 (96.9) | 82 (96.5) | 98 (98) | 84 (97.7) |
| Intradialytic hypotension (patients, %) | 5 (5.2) | 4 (4.7) | 5 (5) | 3 (3.5) |
| Ultrafiltration (ml) | 2228.9±634.9 | 2339.3±706.6 | 2244.9±791.3 | 2530.3±922.5 |
| Pre-dialysis systolic pressure (mmHg) | 144.7±21.4 | 143.72±22.37 | 144.69±21.39 | 140.1±23.24 |
| Post-dialysis systolic pressure (mmHg) | 136.77±22.81 | 137.15±24.37 | 135.88±20.96 | 133.05±20.65 |
| Pre-dialysis diastolic pressure (mmHg) | 76.56±13.75 | 75.03±12.88 | 76.39±14.05 | 75.52±14.51 |
| Post-dialysis diastolic pressure (mmHg) | 74.92±13.36 | 73.75±13.97 | 75.12±13.37 | 75.19±13.08 |
| Hb (g/L) | 108.01±19.98 | 108.15±16.98 | 110.01±15.12 | 106.97±16.53 |
| Alb (g/L) | 40.04±4.56 | 37.50±7.23 | 38.84±5.24 | 36.73±5.76 |
| Glu (mmol/L) | 7.11±3.46 | 7.37±5.01 | 7.65±4.38 | 8.09±4.35 |
| Glycated hemoglobin (%) | 5.78±1.10 | 5.83±1.06 | 5.87±1.03 | 6.13±1.22 |
| TC (mmol/L) | 4.21±1.12 | 4.10±1.51 | 4.07±0.94 | 4.03±1.0 |
| TG (mmol/L) | 2.11±1.43 | 1.84±1.12 | 1.95±0.95 | 1.92±0.89 |
| LDL (mmol/L) | 2.4±0.83 | 2.36±0.71 | 2.20±0.69 | 2.35±0.77 |
| HCO_3_^-^ (mmol/L) | 22.89±3.66 | 23.13±3.06 | 23.08±2.86 | 23.12±3.96 |
| K^+^ (mmol/L) | 4.34±0.89 | 4.08±0.83 | 4.55±1.03 | 4.06±0.92 |
| Ca (mmol/L) | 2.22±0.42 | 2.07±0.27 | 2.19±0.46 | 2.09±0.29 |
| P (mmol/L) | 1.86±0.61 | 1.59±0.53 | 1.94±0.68 | 1.87±0.80^*^ |
| TSAT (%) | 27.19±11.37 | 34.19±17.65 | 27.21±14.02 | 34.45±19.02 |
| Ferritin (ng/ml) | 159.23 (41.66，438.88) | 310.0 (85.5，659.5) | 113.3 (45.8，435.3) | 155.0 (57.4，593.0) |
| iPTH (pg/ml) | 348.87±295.52 | 335.0±253.55 | 365.39±284.83 | 352.27±284.98 |
| 25-(OH)-D3 (mIU/L) | 16.09±14.51 | 24.47±19.79 | 14.75±7.93 | 19.03±12.64 |
| CRP (mg/dl) | 1.2 (0.5, 3.79) | 1.41 (0.5，6.46) | 0.68 (0.5，4.46) | 2.74 (0.72，7.34) |
| β_2_-MG (mg/L) | 26.3 (8.57，36.44) | 28.54 (8.51，37.6) | 21.39 (8.97，32.65) | 24.4 (7.8，34.15) |
| BNP (pg/ml) | 1377 (585.75，4067.25) | 2305.5 (320.5，5602.25) | 1324 (388.75，4701.5) | 1838.5 (764.5，6579.75) |
| spKt/V | 1.53±0.50 | 1.62±0.49 | 1.55±0.50 | 1.50±0.51 |

^*^Compared to 96 weeks in the treatment group, *p*=0.019
